# Coal Flow Foreign Body Classification Based on ESCBAM and Multi-Channel Feature Fusion

**DOI:** 10.3390/s23156831

**Published:** 2023-07-31

**Authors:** Qiqi Kou, Haohui Ma, Jinyang Xu, He Jiang, Deqiang Cheng

**Affiliations:** 1 School of Computer Science and Technology, China University of Mining and Technology, Xuzhou 221116, China; kouqiqi@cumt.edu.cn; 2 School of Information and Control Engineering, China University of Mining and Technology, Xuzhou 221116, China; mahaohui163@163.com (H.M.); kd04161564@cumt.edu.cn (J.X.); jianghe@cumt.edu.cn (H.J.)

**Keywords:** multiple channels, features fusion, attentional mechanism, foreign body classification

## Abstract

Foreign bodies often cause belt scratching and tearing, coal stacking, and plugging during the transportation of coal via belt conveyors. To overcome the problems of large parameters, heavy computational complexity, low classification accuracy, and poor processing speed in current classification networks, a novel network based on ESCBAM and multichannel feature fusion is proposed in this paper. Firstly, to improve the utilization rate of features and the network’s ability to learn detailed information, a multi-channel feature fusion strategy was designed to fully integrate the independent feature information between each channel. Then, to reduce the computational amount while maintaining excellent feature extraction capability, an information fusion network was constructed, which adopted the depthwise separable convolution and improved residual network structure as the basic feature extraction unit. Finally, to enhance the understanding ability of image context and improve the feature performance of the network, a novel ESCBAM attention mechanism with strong generalization and portability was constructed by integrating space and channel features. The experimental results demonstrate that the proposed method has the advantages of fewer parameters, low computational complexity, high accuracy, and fast processing speed, which can effectively classify foreign bodies on the belt conveyor.

## 1. Introduction

Energy is the foundation and support of a country’s prosperity and sustainable economic development, and coal occupies an important position in the subdivision of energy. As the main equipment of coal transportation, the running state of the belt conveyor directly affects the production efficiency of coal. Foreign bodies such as large gangue and bolt in the coal flow not only cause belt scratching and tearing, but also cause problems such as stacking coal, plugging coal, and other problems [1,2,3].

The complex environment of an underground coal mine also challenges traditional target detection and recognition methods [4,5]. With the rapid development of computer vision technology, convolutional neural networks (CNNs) have been widely applied in many fields [6,7,8,9,10] by virtue of their powerful feature extraction ability. In recent years, some scholars have also begun to apply CNNs to the safe mining and transportation of coal. For example, based on the VGG16 network [11,12] and transfer learning, Pu et al. [13] established a foreign body classification model. By designing and improving the LeNet-5 network, as well as the training of 20,000 pictures of foreign bodies in a non-production environment, Su et al. [14] realized the recognition of foreign bodies. On the basis of multispectral imaging and CNNs, Hu et al. [15] optimized the network hyperparameters by Bayes algorithm according to the features of the foreign body image, which also realized the recognition of coal and gangue. Subsequently, by using residual structure and multi-channel feature fusion, studies have [16] established the foreign body classification network of coal belt and achieved remarkable results. Attention mechanism [17,18,19,20], which can selectively focus on some useful features while ignoring others, quickly attracted the attention of scholars as soon as it was developed. At present, attention mechanism has been widely used to further improve the performance of network models. By using the interdependence between feature maps to update the original data, Hu et al. [21] proposed the SENet network model, which can effectively enhance the importance of useful features. On the basis of SENet, Wang et al. [22] proposed ECANet, which obtains more accurate attention by summarizing cross-channel information through a one-dimensional convolution layer. By constructing two sub-modules to combine channel attention with spatial attention, the convolutional block attention module (CBAM) proposed by Woo et al. [23] can obtain more comprehensive and reliable attention information. However, the existing methods of foreign body classification in coal flow still have the following defects: high complexity, heavy computation, low precision, and poor real-time performance, which are not suitable for deployment in the edge intelligent terminal with high requirements.

To solve the above problems, a coal flow foreign body classification network based on ESCBAM and multi-channel feature fusion was constructed. In the proposed network, the key contributions can be summarized as follows: (1) A multi-feature fusion strategy was designed, which can improve the utilization rate of features and the network’s learning ability of detail information. (2) By using the depthwise separable convolution and improved residual network structure as the fundamental feature extraction unit, an information fusion network was constructed to reduce the computational amount. (3) Based on spatial attention and channel attention, and inspired by CBAM network, this paper proposes an improved attention mechanism by using the optimized ECANet and Sam module in ULSAM [24]. To simplify, the improved attention mechanism was named as ESCBAM. For the proposed ESCBAM, it not only can improve the expressive power of network features, but also realize multi-scale feature learning by establishing nonlinear dependence between feature graphs. Thus, the understanding ability of image context can also be enhanced. (4) For experimental evaluation, the proposed method has the advantages of fewer parameters, low computational complexity, high classification accuracy and fast processing speed, which can effectively classify foreign bodies on the belt and has great practical application value.

The remaining sections are organized as follows. Section II briefly reviews the related work. Section III describes the proposed network and its technical essentials. Section IV presents the extensive experimental results. Conclusions and future work are given in Section V.

## 2. Related Work

### 2.1. Depthwise Separable Convolution

Depthwise separable convolution [25,26] is a plug-and-play module, which consists of a depthwise convolution and pointwise convolution, and has been widely used in the convolutional neural network model structure.

**Figure 1:**
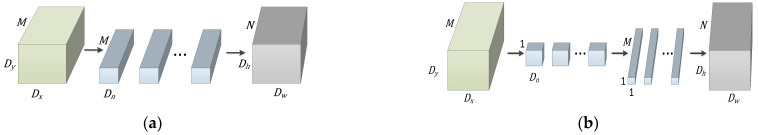
Standard convolution and depthwise separable convolution. (**a**) Standard convolution; (**b**) Depthwise separable convolution.

As shown in Figure 1, given the same input as the standard convolution, after two steps of sequential operation, the output is the same as the result of the standard convolution, but the computation cost is reduced, and the requirements of lightweight parameters and computation amount can be achieved. *M* and *N* denote the number of input and output characteristic channels, respectively. $D_{x}$ and $D_{y}$ are the length and width of the input feature, and $D_{n}$ is the size of the convolutional kernel, whereas $D_{h}$ and $D_{w}$ represent the length and width of the output feature, respectively.

As can be seen from Figure 1a, the computational amount of a standard convolution for *N* convolutional kernels is given by:

(1) $$D_{n}\times D_{n}\times N\times M\times D_{w}\times D_{h}$$

In contrast, the specific structure of a depthwise separable convolution is shown in Figure 1b, and its computation can be expressed by the following formulas:

(2) $$D_{n}\times D_{n}\times M\times D_{w}\times D_{h}$$

(3) $$N\times M\times D_{w}\times D_{h}$$

Therefore, the ratio of the calculational amount of depthwise separable convolution to standard convolution can be expressed by the following formula:

(4) $$\frac{D_{n}\times D_{n}\times M\times D_{w}\times D_{h}+N\times M\times D_{w}\times D_{h}}{D_{n}\times D_{n}\times N\times M\times D_{w}\times D_{h}}$$

As can be seen from Formula (4), with the increase in the number of convolutional kernels, the computational amount of depth separable convolution is significantly lower than that of the standard convolution. The simplified result of the Formula (4) can be given by:

(5) $$\frac{1}{N}+\frac{1}{D_{n}^{2}}$$

### 2.2. CBAM

**Figure 2:**
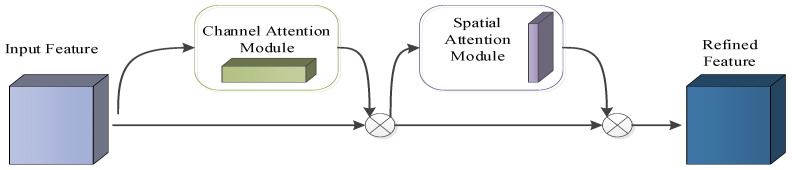
The structure of the CBAM.

As a lightweight module and giving a feature map first, a CBAM module can serialize the attention feature map information in both channel and spatial dimensions. Subsequently, to produce the final feature map, it multiplies the two kinds of feature map information with the original input feature map for adaptive feature correction. The structure diagram of CBAM model is shown in Figure 2.

**Figure 3:**
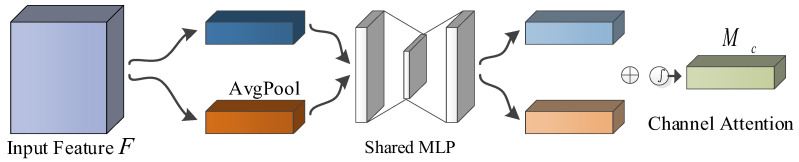
The structure of the channel attention.

In the CBAM, the channel attention mechanism generates the channel attention feature map according to the channel relationship between features. Each channel is considered as a feature detector to compress the feature dimension, and the combination of maximum pooling and average pooling can improve the feature performance of the network. The structure of the channel attention mechanism model is shown in Figure 3.

Firstly, the two feature maps $F_{avg}^{c}$ and $F_{max}^{c}$ are spliced by multi-layer perceptron, in which the multi-layer perceptron contains a hidden layer. Then, the splicing results are sent to the full connection layer, and finally generates the channel feature diagram $M_{c}\in R^{c\times 1\times 1}$ through the processing of the activation function. To reduce the number of parameters, the activation size of the hidden layer is set to $R^{c/r\times 1\times 1}$, where *r* is the reduction ratio. The channel feature diagram can be computed by the Formula (6):

(6) $$M_{c}(F)=\sigma (MLP(AvgPool(F)+MLP(MaxPool(F))))=(W_{1}(W_{0}(F_{avg}^{c})+W_{1}(W_{0}(F_{\max}^{c})))$$

where $W_{0}$ and $W_{1}$ are the weights, respectively, and shared for both inputs.

**Figure 4:**
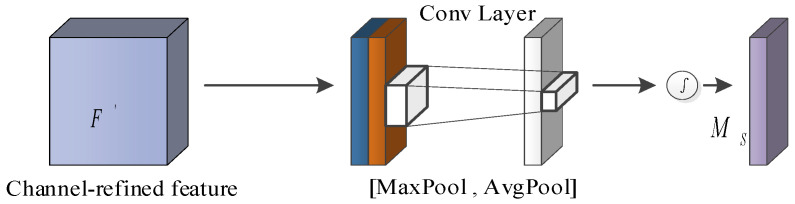
The structure of the spatial attention.

The spatial attention mechanism generates spatial attention feature maps through spatial relationships among features, and its model structure is shown in Figure 4.

As shown in Figure 4, the input feature graph $F^{’}$ is processed by maximum pooling and average pooling to generate two feature graphs $F_{\max}^{S}\in R^{1\times H\times W}$ and $F_{avg}^{c}\in R^{1\times H\times W}$. After the two feature graphs are spliced, the spatial feature graph $M_{S}(F)\in R^{H\times W}$ is generated through the convolution layer, and can be computed by the following formula:

(7) $$M_{S}(F)=\sigma (f^{7\times 7}([AvgPool(F^{’});MaxPool(F^{’})]))=\sigma (f^{7\times 7}([F_{avg}^{S};F_{\max}^{S}]))$$

## 3. Proposed Method

**Figure 5:**
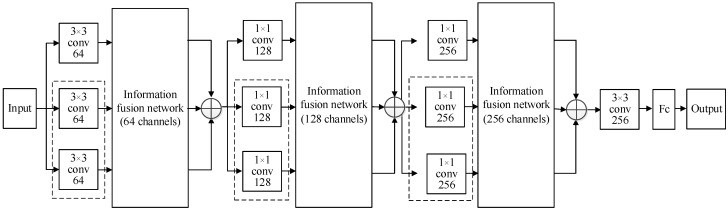
The structure of the proposed network.

**Figure 6:**
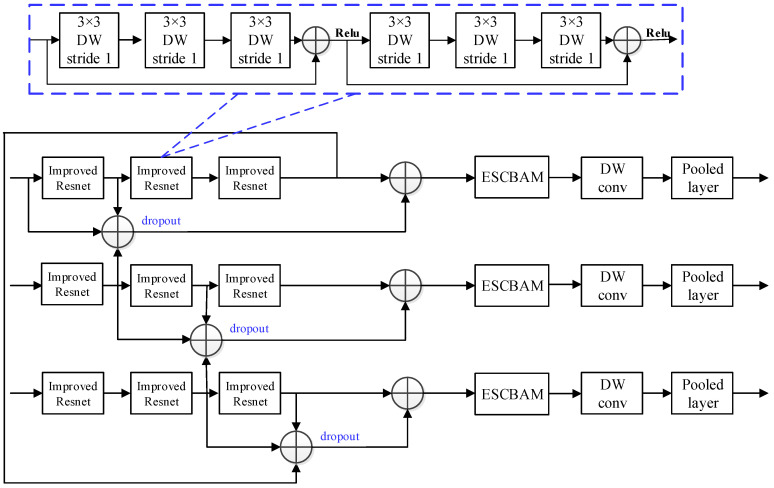
The structure of information fusion network.

In view of the problems existing in a coal flow foreign body classification network, such as large amount of network calculation, poor real-time performance, low recognition accuracy, and not being suitable for deployment in edge intelligent terminal, a novel network based on ESCBAM and multichannel feature fusion is proposed in this paper. The overall structure of the proposed coal flow foreign body classification network is shown in Figure 5 and Table 1. In addition, the detailed structure of the information fusion network in Figure 5 is also presented in Figure 6.

As can be seen, the multi-channel feature fusion network is firstly used to fully integrate the independent feature information between each channel, which can improve the network’s learning ability of detailed information and improve the utilization rate of features. Then, by constructing the information fusion network and using the depthwise separable convolution as well as residual network structure as the basic feature extraction unit, the proposed network can effectively reduce the computational amount while maintaining excellent feature extraction capability during the feature extraction stage. Finally, a novel ESCBAM attention mechanism was constructed based on the idea of integrating space and channel features. The ESCBAM attention mechanism uses two one-dimensional fast convolutions to fuse the feature graphs after average pooling and maximum pooling, which can improve the feature performance of the network. Moreover, the nonlinear dependence relationship between feature maps is captured by different attention maps of two different subspaces to realize multi-scale feature learning and enhance the understanding ability of image context.

### 3.1. Multi-Channel Feature Fusion Network

As shown in Figure 5, multi-channel feature fusion network uses the improved residual structure as the basic feature extraction unit, which is divided into two stages: feature extraction and image classification. In the feature extraction stage, three information fusion networks with different channel numbers are constructed, and each information fusion network contains three improved residual networks. In addition, each improved residual network contains two residual blocks. Finally, the output information of the three improved residual networks is fused.

In the image classification stage, a softmax loss function is adopted. The training set is ${\left\{x_{i}\right\}}_{i=1}^{N}$ and its corresponding label category set is ${\left\{c_{i}\right\}}_{i=1}^{N},c_{i}\in \{1,2,\ldots ,c\}$. Then, the loss function $l_{soft\max}$ can be given by:

(8) $$l_{soft\max}=-\frac{1}{N}\sum_{i=1}^{N}\log(\frac{e^{z_{i}^{c}}}{e^{z_{i}^{c}}+\sum_{j=1,j\neq c}^{c}e^{z_{i}^{j}}})$$

(9) $$z_{i}^{c}={\left(W^{{\left(M\right)}_{c}}\right)}^{T}\cdot X_{i}^{\left(M-1\right)}$$

where $X_{i}$ represents the *i* training sample, *N* denotes the number of training sets, and *c* is the number of training data categories. $W^{{\left(M\right)}_{c}}$ represents column *c* of the parameters of the last layer, and $X_{i}^{\left(M-1\right)}$ denotes the feature expression of the previous layer. In the process of model training, the network parameter $z=\{z_{1},z_{2},\ldots ,z_{c}\}$ is obtained through the gradient descent algorithm, so as to obtain the optimal solution of the loss function. In addition, to avoid overfitting problems during model training, the loss function is further processed by threshold processing: ${\tilde{l}}_{soft\max}=|l_{soft\max}-b|+b$. Herein, ${\tilde{l}}_{soft\max}$ indicates the loss function after threshold processing, and *b* indicates the preset threshold.

### 3.2. Information Fusion Network

As can be seen from Figure 5, the proposed network in this paper contains three information fusion networks, whose channels are 64, 128, and 256, respectively. In addition, to reduce the parameter number and computational cost of the information fusion network, the depthwise separable convolution is used to replace the standard convolution in the residual network to improve the initial residual network. In the information fusion network, the input information is sent into three improved residual networks for further feature extraction. Each improved residual network contains two residual blocks, and each residual block contains three convolutional kernels with size 3 × 3 and step size 1. Finally, the three obtained features are processed by the ESCBAM attention mechanism, convolutional kernel with size 3 × 3 and step size 1, and pooling layer with size 3 × 3, respectively.

The detailed structure of the information fusion network is presented in Figure 6. The improved residual network contains two identical residual blocks, and the specific structure is shown in the blue dotted line box of the Figure 6. In addition, to avoid overfitting caused by the excessive number of parameters, we further dropout the fused features to discard some redundant features, as shown in the blue font in the Figure 6.

### 3.3. ESCBAM Attention Mechanism

**Figure 7:**
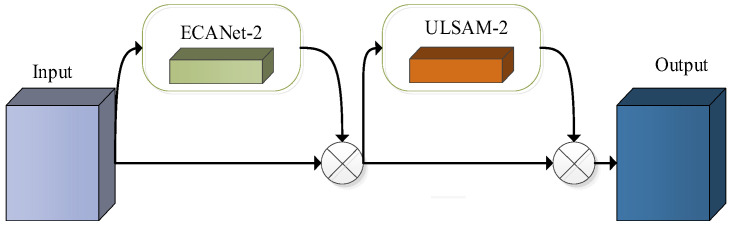
The structure of the ESCBAM.

To further increase the feature representation ability of the proposed network model and minimize the number of parameters and computation, an improved attention mechanism model ESCBAM was also proposed, whose overall structure is presented in Figure 7.

As can be seen from Figure 7, the proposed ESCBAM network mainly consists of two modules, namely ECANet-2 and ULSAM-2. The ECANet [22] is a lightweight channel attention mechanism model, which uses a local cross-channel interaction strategy without dimensionality reduction that can be realized by fast one-dimensional convolution. In this way, it can effectively overcome the paradox of trade-off between performance and complexity.

**Figure 8:**
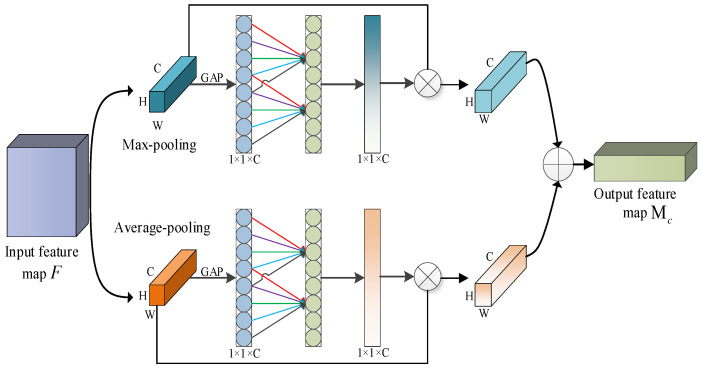
The structure of the ECANet-2.

Herein, the ECANet-2 used in the ESCBAM adopts two fast one-dimensional convolutions to replace the multi-layer perceptron in CBAM. Moreover, it combines the maximum pooling and average pooling feature graphs to reduce the computational load of the model. The detailed structure of the improved ECANet-2 is shown in Figure 8. The convolutional kernel size *k* in the ECANet-2 is set to 5, and its output feature can be expressed by the following formula:

(10) $$M_{C}=M_{avg}+M_{\max}$$

where $M_{avg}$ represents average pooling attention feature map, and $M_{max}$ represents maximum pooling attention feature map.

**Figure 9:**
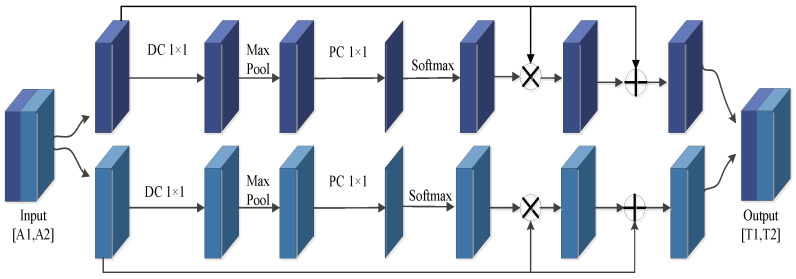
The structure of the ULSAM-2.

For the ULSAM attention mechanism model proposed by Saini et al. [26], it deduces different attention feature maps for each feature subspace, which can realize multi-scale feature representation. Herein, the spatial attention mechanism in the ESCBAM adopts the ULSAM-2 structure, and its specific structure is shown in Figure 9.

ULSAM-2 divides the feature mapping into two different subspaces, and then calculates each subspace separately. The formula is as follows:

(11) $$T_{n}=soft\max(PW(\max pool(DW^{1\times 1}(A_{n}))))$$

(12) $${\tilde{T}}_{n}=(T_{n}⊗A_{n})⊕A_{n}$$

(13) $$\tilde{T}=f([{\tilde{T}}_{1},{\tilde{T}}_{2}])$$

where $A_{n}$ denotes the input feature maps of different subspaces, and $\tilde{T}$ represents the output feature map of ULSAM-2. The input feature map of ULSAM-2 is divided into $A_{1}$ and $A_{2}$, and formulas (11) and (12) represent the operation flow of one of $A_{n}$.

As can be seen from Figure 9, $A_{n}$ undergoes a depthwise separable convolution of 1 × 1, maximum pooling of 3 × 3, and point-by-point convolution involving a convolutional kernel in turn. Then, after processing by softmax loss function, the subspace output feature map $T_{n}$ is obtained. Subsequently, it is operated by ⊗ and ⊕ with the subspace input feature map An, respectively, to get ${\tilde{T}}_{n}$. ⊗ represents matrix multiplication and ⊕ represents matrix superposition. Finally, ${\tilde{T}}_{1}$ and ${\tilde{T}}_{2}$ are combined to obtain the output feature map $\tilde{T}$, and *f* represents the splicing operation. In this way, the ULSAM-2 model can effectively capture the nonlinear dependence relationship between different feature maps by forming different attention mappings for two different subspaces. In addition, the multi-scale feature learning can also be realized, and the understanding ability of image context is enhanced, and the cross-channel information is integrated through different feature mapping subspaces.

## 4. Experimental Results

In this section, to validate the superiority of the proposed method, comprehensive comparative evaluations with the existing networks were conducted on two public standard databases Cifar10 and Cifar100, as well as the real-world applications, that is, the mining belt conveyor coal flow dataset CUMT-BelT. The experiments in this paper were carried out on Ubuntu 20.04.2, Intel(R) Core(TM) I9-10980X@3.0ghz, GPU NVIDIA GeForce RTX3090 (Santa Clara, CA, USA), video capacity 24 GB, and memory 64 GB. CUDA was version 11.1 and Pytorch framework was version 1.8. In addition, the initial learning rate of the proposed network was set as 0.0001, 80 times per iteration, and the learning rate was multiplied by 0.2, for a total of 240 iterations.

### 4.1. Datasets and Experimental Setup

All the experimental tests in this paper were conducted on three datasets, including two public datasets and one self-built dataset. Public datasets Cifar10 and Cifar100 were selected, and the pictures of self-built datasets were from the real production environment under the mine.

The Cifar10 [27] dataset consisted of a total of 60,000 color RGB images of 10 categories of aircraft, cars, birds, cats, deer, dogs, frogs, horses, boats, and trucks with the size of 32 × 32. There were 6000 images for each category, which were divided into 5000 training images and 1000 test images.

The Cifar100 [27] dataset was also composed of 60,000 32 × 32 color RGB images, with a total of 100 categories, each of which contained 600 images, divided into 500 training set images and 100 test set images. In addition, the 100 categories were divided into 20 super-categories, and each image carried a “fine” label (i.e., the category to which it belonged) and a “rough” label (i.e., the super-categories to which it belonged).

**Figure 10:**
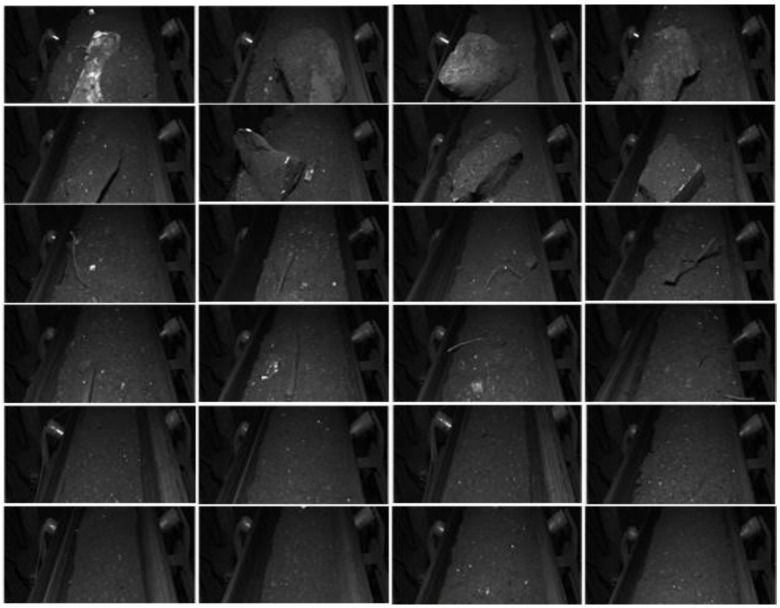
Display of belt conveyor coal flow dataset CUMT-BelT.

The mining belt conveyor coal flow dataset CUMT-BelT was collected from the transport environment of the belt under the mine. The exposed benchmark dataset can be downloaded from the following address: https://github.com/CUMT-AIPR-Lab/CUMT-AIPR-Lab (accessed on 7 June 2023). The dataset contains 6000 pictures in total, which are divided into 3 categories: large gangue, bolt, and normal sample. Each category consists of 2000 images, with 1600 images allocated for training and 400 images for testing. A portion of the dataset is depicted in Figure 10. As evident from the illustration, the pictures in the first and second row are the sample pictures of large gangue, characterized by their substantial size and weight. Once the coal drop port is blocked in the process of coal flow transmission, it is easy to cause accidents such as coal piling, coal blocking, and even belt tearing. The pictures in the third and fourth rows are the samples of the bolt, which is sharp and slender, and it is very easy to scratch and tear the belt in the process of coal flow transmission. The last two lines are normal coal flow pictures.

### 4.2. Experimental Results and Discussion

In this subsection, to explore the influence of attention mechanisms on the classification effect of the proposed network, SENet [21], ECANet [22], CBAM [23], and ESCBAM were embedded into the proposed network, respectively, for comparative experiments, and the embedded positions of the four attention mechanisms were all the same. The comparison results of classification accuracy and computational amount on Cifar10 dataset and mining belt conveyor coal flow dataset are shown in Table 2.

As can be seen from Table 2, the network model incorporating CBAM and ESCBAM modules exhibited higher classification accuracy than the network with SENet and ECANet modules for both the Cifar10 and the mining belt conveyor coal flow dataset. However, it is worth noting that the computational cost of the former was also higher compared to SENet and ECANet across the board. The main reason is that the CBAM and ESCBAM modules both incorporate channel attention and spatial attention, whereas SENet and ECANet contain only spatial attention, so this is what we expected. Moreover, the classification accuracy of the network with ESCBAM on Cifar10 was not only 0.2% higher than that of the network with CBAM, but also 0.2% higher on the mining belt conveyor coal flow dataset, and the computational amount was also reduced by 0.17 G. Those results fully indicate that the constructed ESCBAM attention mechanism can not only improve the classification accuracy, but also reduce the computational amount, making it possible to be deployed in the edge intelligent terminal with high requirement of real-time mine target recognition in the near future.

**Figure 11:**
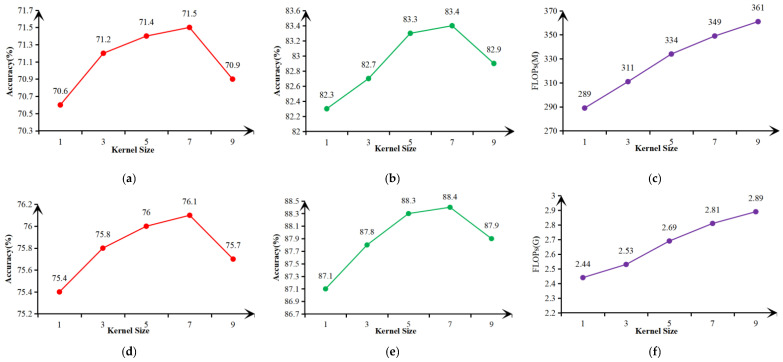
The effect of different convolutional kernel sizes on network performance. (**a**) Accuracy of MobileNetV2 with different *k* of ESCBAM on Cifar100; (**b**) Accuracy of MobileNetV2 with different *k* of ESCBAM on CUMT-BelT; (**c**) Computation of MobileNetV2 with different *k* of ESCBAM; (**d**) Accuracy of the proposed network with different *k* of ESCBAM on Cifar100; (**e**) Accuracy of the proposed network with different *k* of ESCBAM on CUMT-BelT; (**f**) Computation of the proposed network with different *k* of ESCBAM.

To explore the influence of one-dimensional fast convolutional kernel’s size in the ECANet-2 of ESCBAM module on the performance of attention mechanism, five convolutional kernels with different sizes (1, 3, 5, 7, 9) were selected empirically in this paper, and comparative experiments were conducted on the mining belt conveyor coal flow dataset and the Cifar100 dataset. The overall network structure was MobileNetV2 [28] and the network proposed in this paper. The experimental results are shown in Figure 11 and Table 3.

It can be seen from Figure 11 and Table 3 that as the convolutional kernel size increased, so did the computational amount of the corresponding network. However, it can also be observed that the classification accuracy did not always increase with the increase of the size of the convolutional kernel, but decreased after reaching a certain peak value. Therefore, the selection of the convolutional kernel size will directly affect the classification accuracy of the proposed network. For the MobileNetV2 network, the classification accuracy on Cifar100 and mining dataset were both the highest when *k* = 7, which was 0.1% higher than that when *k* = 5, but it increased 15 M in FLOPs. On the whole, although the classification effect when *k* = 7 was slightly better than that when *k* = 5, the amount of computation increased significantly. As for the network proposed in this paper, although its classification accuracy was also the highest on Cifar100 and mining dataset when *k* = 7, and was 0.1% higher than that when *k* = 5, its computational amount was also significantly increased by 0.17 G than that when *k* = 5. Moreover, there was a significant decline in classification accuracy in both datasets when k = 9. Therefore, compared with 0.1% improvement in classification accuracy, the reduction in computational amount worth weighing and paying more attention to. Hence, *k* = 5 was finally selected as the size of the convolutional kernel of ESCBAM module in this paper.

To further verify the validity and complexity of our network model and ESCBAM attention mechanism, 32 networks in 8 categories (i.e., our proposed network, MobileNetV2 [28], ResNet50 [29], ResNet34 [29], GoogleNetV3 [30], ResNeXt50 [31], ShufflenetV2 [32], and Yang et al. [33] with four different attention mechanisms, none, ECANet, CBAM, and ESCBAM, respectively) were tested on the Cifar10, Cifar100, and mining belt conveyor coal flow datasets, respectively. In addition, the four indexes of parameter number, FLOPs, FPS, and accuracy were adopted as their performance evaluation indexes, and the comparison results are listed in Table 4. The best results for the 4 different attention mechanisms of each category are shown in blue bold, and the best results achieved across all 32 networks are shown in black bold.

As can be seen, the same classification network with different attention mechanisms had great differences in the four indicators, and different classification networks with the same attention mechanisms also had great differences in performance. On the whole, the network model using attention mechanism had better classification accuracy than the networks model without the attention mechanism. Moreover, the ESCBAM proposed in this paper achieved the most remarkable performance of all the attention mechanisms.

For the ResNet50 and ResNet34 networks, compared with networks without the attention mechanism on the Cifar10 dataset, the accuracy of networks using ECANet, CBAM, and ESCBAM increased by 1.5%, 1.9%, 2.2% and 2.2%, 3.0%, 3.1%, respectively. The accuracy of the corresponding networks in Cifar100 dataset increased by 1.5%, 2.2%, 2.4% and 2.1%, 2.6%, 2.8%, as well as in mining belt conveyor coal flow dataset by 2.4%, 3.0%, 3.1% and 1.7%, 2.2%, 2.3%, respectively. Therefore, we can conclude that the accuracy of the network can be improved in both the public dataset and the mining belt conveyor coal flow dataset, indicating that the ESCBAM attention mechanism proposed in this paper has strong generalization. In addition, although the proposed network is relatively not optimal in terms of parameters, FLOPs and FPS, its accuracy is the highest among other attention mechanisms, and it also achieves better performance than the network with CBAM in terms of parameters, FLOPs, and FPS. Analogously, the above similar conclusions can also be found from the results of GoogleNetV3 and Yang et al.

For the ResNeXt50 network, compared with the network using ECANet or the network without the attention mechanism, the classification accuracy of networks using ESCBAM were significantly improved on all the datasets. Compared with networks using CBAM, the classification accuracy of our network using ESCBAM on the three datasets decreased by 0.1%, remained unchanged, and increased by 0.1%, respectively, indicating that the average classification accuracy of these two methods remains unchanged. However, it is worth mentioning that its average classification accuracy remains the same, but its amount of computation decreased by 0.16 G, its number of parameters decreased by 1.7 M, and the FPS increased by 2.

For the ShufflenetV2 and MobileNetV2 networks with fewer parameters and lower FLOPs, compared with the network without the attention mechanism on the Cifar10 dataset, the accuracy of networks using ECANet, CBAM, and ESCBAM increased by 2.2%, 3.0%, 3.3% and 2.1%, 2.7%, 3.0%, respectively. In addition, the accuracy of the corresponding networks in the Cifar100 dataset increased by 1.8%, 2.6%, 2.8% and 1.5%, 2.2%, 2.5%, as well as in the mining belt conveyor coal flow dataset by 2.5%, 3.0%, 3.2% and 2.3%, 2.8%, 2.9%, respectively. Therefore, through the above two groups of experimental results from the ShufflenetV2 and MobileNetV2 networks, it has again proved that the ESCBAM attention mechanism proposed in this paper has strong generalization and portability.

For the network proposed in this paper, compared with network using ECANet or network without the attention mechanism, the proposed network using ESCBAM achieved the highest classification accuracy on the Cifar10, Cifar100, and mining belt conveyor coal flow datasets. Compared with the network using CBAM, the classification accuracy of our network using ESCBAM increased by 0.2% on all three datasets. Furthermore, its computational load also reduced by 0.17 G, the number of parameters reduced by 1.3 M, and the FPS increased by 4. Therefore, we can conclude that the novel ESCBAM can not only improve the classification accuracy, but also reduce the number of parameters and the computational amount, indicating that our ESCBAM is not only suitable for other classification models, but also suitable for the classification model based on multi-channel feature fusion proposed in this paper.

In summary, through the above detailed experimental comparison and analysis, it is proven that the proposed foreign body classification model of coal flow based on ESCBAM and multi-channel feature fusion has the advantages of fewer network parameters, low computational complexity, high classification accuracy, and fast processing speed, which can effectively classify foreign bodies on the coal belt, thus improving the transportation efficiency of the mine coal belt.

## 5. Conclusions

In this paper, a coal flow foreign body classification network based on ESCBAM and multi-channel feature fusion is proposed. Firstly, a multi-channel feature fusion strategy was designed, which improved the network’s learning ability to detailed information and feature utilization. Subsequently, by using the depthwise separable convolution and improved residual network structure as the basic feature extraction unit, and then constructing the information fusion network, the computational amount of the proposed network was effectively reduced, and the remarkable feature extraction capability was also maintained. Finally, based on the idea of integrating space and channel features, a novel ESCBAM attention mechanism with strong generalization and portability was constructed and further embedded into the information fusion network. Comprehensive experimental results of three databases demonstrate that the proposed network can achieve high classification accuracy and fast processing speed while enjoying a fewer network parameters and lower computational complexity.

In future work, the multiplex attention multiplexing mechanism and hierarchical feature-guided attention mechanism will be investigated and used to try to optimize the proposed method to further improve the accuracy of coal flow foreign body classification.

## Figures and Tables

**Table 1:** The detailed structure of the proposed network.

Input	Information Fusion Network (64 Channels)	Information Fusion Network (64 Channels)	Information Fusion Network (64 Channels)	Output
3 × 3, 64	{3 × 3, 64, DW} × 21,stride1 {1 × 1, 128} × 1,stride1 3 × 3 max pool, stride2	{3 × 3, 128, DW} × 21,stride1 {1 × 1, 256} × 1, stride1 3 × 3 max pool, stride2	{3 × 3, 256, DW} × 21, stride1 {1 × 1, 256} × 1, stride1 3 × 3 max pool, stride2	3 × 3, 256 average pool fc soft max

**Table 2:** Accuracy and computational cost comparison of networks with different attention mechanisms.

Method Tested	Cifar10 (%)	CUMT-BelT (%)	FLOPs
Our + SENet	94.4	86.8	2.65 G
Our + ECANet	95.5	87.7	2.48 G
Our + CBAM	96.1	88.1	2.86 G
Our + ESCBAM	96.3	88.3	2.69 G

**Table 3:** Accuracy and computational cost comparison of networks with different convolutional kernel sizes.

Method Tested	Convolutional Kernel Size of ESCBAM	Cifar100 (%)	CUMT-BelT (%)	FLOPs
MobileNetV2	k = 1	70.6	82.3	289 M
	k = 3	71.2	82.7	311 M
	k = 5	71.4	83.3	334 M
	k = 7	71.5	83.4	349 M
	k = 9	70.9	82.9	361 M
Ours	k = 1	75.4	87.1	2.44 G
	k = 3	75.8	87.8	2.53 G
	k = 5	76.0	88.3	2.69 G
	k = 7	76.1	88.4	2.81 G
	k = 9	75.7	87.9	2.89 G

**Table 4:** Comparison of parameters, accuracy, FLOPs, and FPS of different networks with different attention mechanisms.

Networks	Attention Mechanism	Params (M)	Cifar10 (%)	Cifar100 (%)	CUMT-BelT (%)	FLOPs	FPS
ResNet50	/	27.2	93.5	73.4	84.4	4.12 G	67
	ECANet	30.6	95.0	74.9	86.8	4.77 G	63
	CBAM	34.7	95.4	75.6	87.4	4.93 G	60
	ESCBAM	32.5	**95.7**	**75.8**	**87.5**	4.84 G	62
ResNet34	/	22.3	92.1	71.1	82.9	3.78 G	69
	ECANet	24.1	94.4	73.2	84.6	4.01 G	67
	CBAM	29.3	95.1	73.7	85.1	4.28 G	62
	ESCBAM	26.9	**95.2**	**73.9**	**85.2**	4.18 G	65
GoogleNetV3	/	6.8	88.2	69.7	81.3	1.80 G	113
	ECANet	8.7	90.6	71.6	83.2	1.97 G	107
	CBAM	10.1	91.0	72.5	83.8	2.14 G	97
	ESCBAM	9.2	**91.4**	**72.8**	**84.0**	2.01 G	105
ResNeXt50	/	26.8	94.0	73.8	84,6	4.04 G	67
	ECANet	28.9	95.3	75.1	86.9	4.62 G	62
	CBAM	33.8	**95.9**	**75.9**	87.2	4.89 G	58
	ESCBAM	32.1	95.8	**75.9**	**87.3**	4.73 G	60
ShufflenetV2	/	2.4	88.4	68.3	80.1	145 M	141
	ECANet	3.2	90.6	70.1	82.6	166 M	133
	CBAM	4.4	91.4	70.9	83.1	190 M	128
	ESCBAM	3.6	**91.7**	**71.1**	**83.3**	174 M	130
MobileNetV2	/	2.3	88.8	68.9	80.4	274 M	122
	ECANet	3.0	90.9	70.4	82.7	299 M	120
	CBAM	4.3	91.5	71.1	83.2	358 M	115
	ESCBAM	3.5	**91.8**	**71.4**	**83.3**	334 M	116
Yang et al.	/	16.8	93.8	72.9	84.0	3.14 G	79
	ECANet	19.1	94.6	74.3	86.8	3.42 G	75
	CBAM	21.6	94.9	75.1	87.4	3.97 G	68
	ESCBAM	20.2	**95.5**	**75.2**	**87.9**	3.76 G	71
Ours	/	14.1	93.7	73.2	84.2	2.02 G	107
	ECANet	16.3	95.5	75.4	87.7	2.48 G	101
	CBAM	19.7	96.1	75.8	88.1	2.86 G	95
	ESCBAM	18.4	**96.3**	**76.0**	**88.3**	2.69 G	99

## Data Availability

The data may be obtained from the authors upon reasonable request.
